# Prevalence, species distribution and antifungal susceptibility of *Candida albicans* causing vaginal discharge among symptomatic non-pregnant women of reproductive age at a tertiary care hospital, Vietnam

**DOI:** 10.1186/s12879-021-06192-7

**Published:** 2021-06-03

**Authors:** Do Ngoc Anh, Dao Nguyen Hung, Tran Viet Tien, Vu Nhat Dinh, Vu Tung Son, Nguyen Viet Luong, Nguyen Thi Van, Nguyen Thi Nhu Quynh, Nguyen Van Tuan, Le Quoc Tuan, Nguyen Duy Bac, Nguyen Khac Luc, Le Tran Anh, Do Minh Trung

**Affiliations:** 1 Department of Medical Parasitology, Vietnam Military Medical University, Hanoi, Vietnam; 2 Laboratory of Parasitology and Medical Mycology, 103 Military Hospital, Hanoi, Vietnam; 3 103 Military Hospital, Vietnam Military Medical University, Hanoi, Vietnam; 4 Department of Epidemiology, Vietnam Military Medical University, Hanoi, Vietnam; 5 Vietnam Military Medical University, Hanoi, Vietnam; 6 Institute of Biomedicine and Pharmacy, Vietnam Military Medical University, Hanoi, Vietnam

**Keywords:** Prevalence, Vulvovaginal candidiasis, Reproductive age women, Antifungal susceptibility

## Abstract

**Background:**

Vaginal candidiasis is frequent in women of reproductive age. Accurate identification *Candida* provides helpful information for successful therapy and epidemiology study; however, there are very limited data from the Vietnam have been reported. This study was performed to determine the prevalence, species distribution of yeast causing vaginal discharge and antifungal susceptibility patterns of *Candida albicans* among symptomatic non-pregnant women of reproductive age.

**Methods:**

Vaginal discharge samples were collected from 462 women of reproductive age in Hanoi, Vietnam between Sep 2019 and Oct 2020. Vaginal swabs from these patients were examined by direct microscopic examination (10% KOH). CHROMagar™ Candida medium and Sabouraud dextrose agar supplemented with chloramphenicol (0.5 g/l) were used to isolate yeast, and species identification was performed using morphological tests and molecular tools (PCR and sequencing). Antifungal susceptibility testing was determined according to the Clinical and Laboratory Standards Institute guidelines (M27-A3 and M27-S4).

**Results:**

The prevalence of vaginal yeast colonization in non-pregnant women was 51.3% of 462 participants. Nine different yeast species were identified. Among these isolates, *C. albicans* (51.37%) was the most frequent, followed by *C. parapsilosis* (25.88%), *C. glabrata* (11.37%), *C. tropicalis* (4.31%), *C. krusei* (3.92%), *C. africana* (1.57%), *Saccharomyces cerevisiae* (0.78%), *C. nivariensis* (1 isolates, 0.39%), and *C. lusitaniae* (1 isolates, 0.39%), respectively. Among *C. albicans,* all 46 isolates were 100% susceptible to micafungin, caspofungin, and miconazole. The susceptibility rates to amphotericine B, 5-flucytosine, fluconazole, itraconazole and voriconazole were 95.65, 91.30, 91.30, 82.61 and 86.95%, respectively.

**Conclusions:**

The prevalence of VVC among symptomatic non-pregnant women of reproductive age in Vietnam was higher than many parts of the world. The high frequency of non-*albicans Candida* species, which were often more resistant to antifungal agents, was a notable feature. Resistance rates of vaginal *C. albicans* isolates to antifungal agents was low. Our findings suggest that continued surveillance of changes in species distribution and susceptibility to antifungals should be routinely screened and treated.

**Supplementary Information:**

The online version contains supplementary material available at 10.1186/s12879-021-06192-7.

## Introduction

*Candida* species are part of the normal flora of the genital tract. In healthy asymptomatic non-pregnant women, these yeasts have been found in 20–30% [1, 2]. Approximately 75% of all women experience at least one episode of vulvovaginal candidiasis (VVC) and 40–50% will have recurrent episodes during their lifetime [2, 3]. Epidemiology of vaginal *Candida* infection suggests that up to 50% of all women would have experienced two or more episodes of VVC by the age of 25 years. This age group with the onset of sexual activity is an important risk factor [4]. The risk factors are believed to be associated with increased rate of VVC including host-related factors such as hyper-estrogenic state (pregnancy, hormone replacement therapy), poorly controlled diabetes, immunodeficiency states, use of antibiotic, treatment with glucocorticoids and genetic predispositions and behavioral factors such as birth control pills, intrauterine device, spermicides and condoms and hygiene habits, tight-fit clothing and sexual behaviour [5].

Epidemiological surveys around the world have indicated that distribution of *Candida* species responsible for VVC in women varies widely among countries, regions and also the study population, and women with vaginal candidiasis are more susceptible to HIV [2, 6]. Traditionally, *Candida albicans,* which is responsible for 85–95% of *Candida* vaginal infections, is the predominant species [7–10]; however*,* the raising frequency of non-*albicans Candida* (NAC) species has reported worldwide, particularly *C. glabrata*, *C. tropicalis*, *C. parapsilosis*, *C. krusei*, *C. dubliniensis*, with *C. glabrata* as the predominant species [8–10]*.* There are many options for the treatment of uncomplicated VVC caused by *C. albicans*. A variety of short-course topical treatments are available, including polyenes, imidazoles, or ciclopirox olamine [11]. In that, the azole antifungals are most usually available in vaginal suppositories and creams [11, 12]. Therapy with the oral azoles, including fluconazole, itraconazole and miconazole, is also possible [11]. In contrast, complicated VVC requires a more prolonged course of therapy and is often difficult to achieve successful results, especially in the patient with VVC caused by NAC [13]. The epidemiological data from previous studies showed that NAC species are more common among complicated VVC than uncomplicated cases and NAC species are often more resistant to antifungal agents [9, 14, 15]. Therefore, identification and antifungal susceptibility testing are necessary for selection of appropriate antifungal therapy and performance of infection control methods to prevent the transmission of these infections [9, 13].

In recent years there have been many publications about identification and antifungal susceptibility of *Candida* causing VVC from different countries in the world as China [16], Iran [7, 17, 18], Japan [19], Lebanon [1], Ethiopia [20] and Italia [21], etc., but very limited data is available on the species distribution of *Candida* isolates in cases of VVC in Vietnam. The epidemiology of antifungal resistance among *C. albicans* in reproductive age women in Vietnam remains poorly reported. Therefore, the aim of this study was to determine the prevalence, species distribution of yeast causing vaginal discharge and antifungal susceptibility pattern of *C. albicans* among symptomatic non-pregnant women of reproductive age.

## Methods

### Study population

In current study, a cross-sectional design was conducted between Oct 2019 and Sep 2020. A total of 462 symptomatic non-pregnant women within the age range of 18–49 years with a clinical picture suggestive of VVC were selected from obstetrics and gynecology clinic at 103 Military Hospital (550 beds, Ha Dong district) in Hanoi city, Vietnam. Patients who were non-married, in pregnancy, outside the childbearing age period or using any systemic or topical antifungal therapy in the previous 2 weeks were excluded from the study.

### Sampling, phenotypic identification of yeast species

The diagnosis of VVC was based on clinical appearance and culturing on CHROMagar™ Candida (CHROMagar Company, Paris, France) plates. A case of the VVC was defined as a patient with symptoms and signs include vulvar itching, vaginal discharge, and had positive results for *Candida* spp. [22]. Clinical samples were taken from each patient’s vagina by obstetrician and gynecologist with sterile cotton-tipped swab. After that, vaginal swabs were transported to the clinical mycology or microbiology laboratory within 2 h for isolation of yeasts. And then, all of vaginal swab specimens were subjected to direct 10% KOH smear examination as well as cultured on CHROMagar™ Candida plates, incubated at 35 °C for 4 days to determine the co-infection rate of yeast species and distinguish between *C. albicans, C. tropicalis, C. glabrata* and *C. krusei* and other yeast species according to the manufacturer’s instructions. All agar plates were evaluated for the yeast growth and colony color every day. The presence of yeasts on agar plates was confirmed by the presence of budding yeast in wet preparations with 0.85% saline. If yeast species were the same, it was considered to be a single isolate. All yeast isolates were initially subcultured on Sabouraud dextrose agar (SDA) (Merck, Germany) supplemented with 0.02% chloramphenicol and kept in tryptic soy broth medium (TSB, Himedia, India) containing 2.5% glucose, 3% peptone, and 20% glyceron at − 80 °C for further use. Germ tube tests in serum at 37 °C for 2–3 h was also used for the differentiation of *C. albicans* and NAC species**.**

### Genomic DNA isolation

Genomic DNA of yeasts was extracted from isolates using a commercial DNA isolation kit (Cat.#51,304, QIAGEN, Germany) according to the manufacturer’s recommendations. After that quality and quantity of extracted DNA was estimated using a NanoDropTM 2000 Spectrophotometer at 260 nm (Thermo Fisher Scientific, USA). The purified DNA was maintained at − 20 °C until used in the PCR.

### Molecular identification of *C. albicans* species complex

*C. albicans* species complex was identified by molecular techniques PCR using the specific primer pair of CR-f (5′-GCTACCACTTCAGAATCATCATC-3′) and CR-r (5′-GCACCTTCAGTCGTAGAGACG-30) (Integrated DNA Technologies, USA) for the hyphal wall protein 1 (HWP1) gene [23]. The amplified PCR products of HWP1 gene were used to distinguish *C. albicans* (941 bp), *C. dubliniensis* (569 bp) and *C. africana* (750 bp) [23]. PCR reaction were performed as previously described [23]. Reference strain used as a control was *C. albicans* (ATCC 90028).

### Determination of NAC based on PCR-RFLP technique

NAC species were identified by PCR-RFLP assays using universal primers (Integrated DNA Technologies, USA) and restriction enzyme *Msp*I as described by previous studies [24, 25]. Total volume of PCR reactions was 30 μl containing 3 μl of DNA solution, 15 μl 2X Master Mix (Cat.# M7505, Promega, USA), 0.75 μl of each primer (0.25 μM) and distilled water up to 30 μl. PCR amplification was carried out with Thermo Mastercycler Gradient (Thermo Fisher Scientific, USA). PCR reaction were carried out as described in previous study [24]. Restriction fragment length polymorphism (RFLP) assay was performed in a total volume of 20 μl containing 10 μl PCR product, 1 μl of *Msp*I enzyme (BioLabs, England), 2 μl of 10X NEbuffer (BioLabs, England) and 7 μl of distilled water. According to the manufacturer’s recommendations, the mix tubes were incubated at 37 °C for 3 h, and then enzyme was completely inactivated by heating at 65 °C for 15 min. The amplified PCR and digestion products were analyzed on 2.0% agarose gels containing 0.5 μg/ml ethidium bromide in 1X TBE buffer for about 1.5 h at 90 V and visualized on UV illumination (UVP, Canada). The PCR products and digestion sizes were determined by a 100 bp size marker (Cleaver, UK). In addition, unknown isolates were subjected to direct DNA sequencing.

### ITS rDNA region sequencing

Amplification of the ITS1 and ITS2 domains of the rRNA gene were performed with the primers ITS5 (5′-GGA AGT AAA AGT CGT AAC AAG − 3′) and NL4 (5′- GGT CCG TGT TTC AAG ACG G − 3′) as previously described [26]. The PCR products of forty strains of yeast were shipped to Apical Scientific Sdn. Bhd (Kembangan 43,300, Selangor, Malaysia) for purification and automatic bidirectional sequencing, using the same PCR primers. ABI 3130 Genetic Analyzer software (SeqScape 2.1) was used to read the sequences. Species identification was accurately confirmed by two-directional sequencing. In total there were 40 fungal cultures sequenced, and all sequences were deposited in GenBank under the accession number MW307689-MW307696, MW307701-MW307705, MW307707-MW307727, MW057251- MW057254, MW057257, and MW055675.

### In vitro antifungal susceptibility test

*C. albicans* isolates were removed from the − 70 °C freezer and revived on a SDA plate at 35 °C for 24 h. Fungal suspensions in saline sulotion of isolates were made and the density of the suspension was adjusted at 0.5 McFarland standards at a wavelength of 530 nm, and diluted to 0.5 × 10^3^ or 2.5 × 10^3^ cells/ml using RPMI 1640 medium. The antifungal susceptibility tests with micafungin (MFG), caspofungin (CAS), amphotericin B (AMB), flucytosine (5-FC), fluconazole (FCZ), itraconazole (ICZ), voriconazole (VCZ), miconazole (MCZ) (Sigma-Aldrich, St. Louis, MO, USA) were performed for 46 *C. albicans* strains using broth microdilution method as described in the clinical and laboratory standard institute (CLSI) document M27-A3 [27]. These strains were selected from all patients who had at least two episode of VVC within a year. Stock solutions of the antifungal agents were prepared in the appropriate solvent according to the CLSI [27]. The final concentration ranges were between 0.015 and 16 μg/mL for micafungin; 0.03 and 16 μg/mL for caspofungin, amphotericin B and miconazole; 0.12 and 64 μg/mL for flucytosine; 0.12 and 64 μg/mL for fluconazole; 0.015 and 8 μg/mL for itraconazole and voriconazole. The minimum inhibitory concentration (MIC) values for all antifungal agents were read after 24 and 48 h of incubation at 35 °C. The MIC values of AMB were visually determined at the lowest concentration of agent that prevents any visible growth (100%). The MICs endpoints of the azole, echinocandins and flucytosine have been defined as the lowest drug concentration at which there is at least a 50% decrease in growth compared to that of the drug-free well. Species-specific clinical breakpoints for isolated *Candida* were classified according to the M27-S4 document [28]. Because breakpoint for miconazole was not defined yet in literature, it is indicated that *C. albicans* is susceptible and resistant at MIC ≤1 μg/mL and MIC ≥8 μg/mL, respectively [29, 30]. Resistance rate is the percentage of isolates resistant to a specific antifungal drug. For quality control, *C. parapsilosis* (ATCC 22019) and *C. albicans* (ATCC 90028) were chosen as reference strains.

### Statistical analyses

All statistical calculations were done using IBM SPSS Statistics for Microsoft Windows, version 20.0 (IBM Corp., Armonk, NY, USA). The sequences of the ITS-1 and ITS-2 regions of yeasts were compared to the available data in the NCBI database, using BLAST guidelines (http://blast.ncbi.nlm.nih.gov/Blast.cgi).

## Results

During the study period, the 462 vaginal samples were collected from vagina of patients with signs and symptoms of vaginal infection. Of them, 237 (51.30%) patients were diagnosed with VVC. Patients had a mean age of 35.51 ± 7.99 years (range, 18–49 years). Women between 30 and 39 years had the highest prevalence rates of VVC (54.67%), while in the age groups 18–29 and 40–49 the prevalence rates were 51.96 and 45.89%, respectively.

**Fig. 1:**
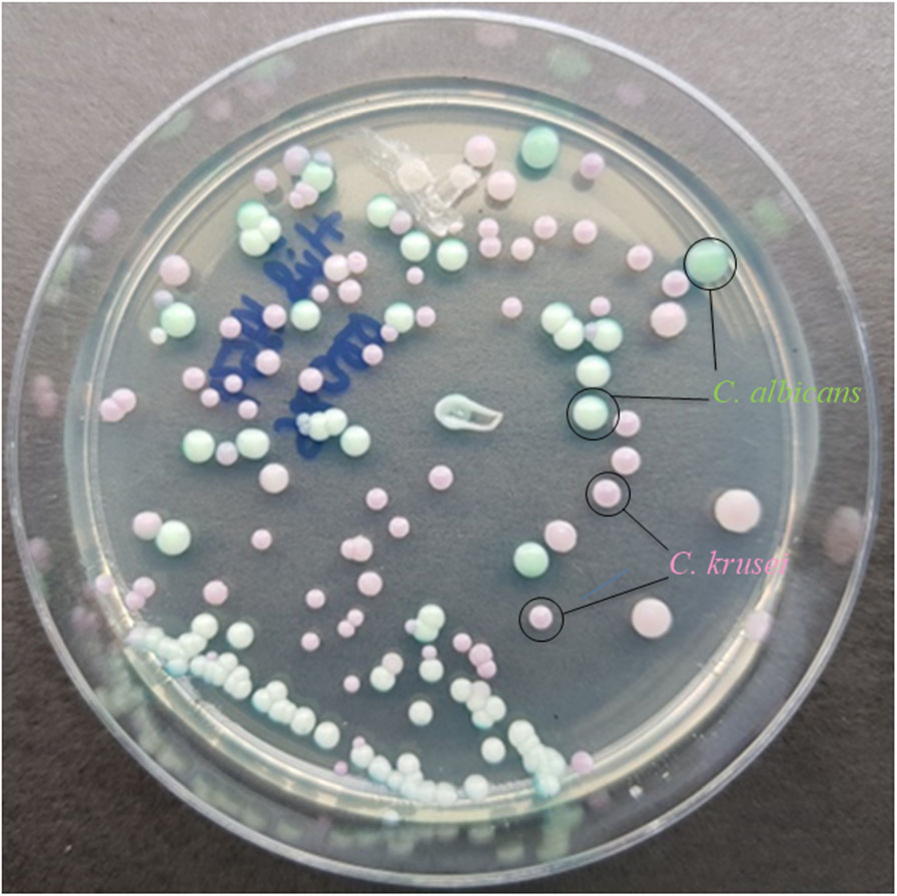
Co-infection of *Candida albicans* and *Candida krusei* in a VVC patient on CHROMagar™ Candida medium

Of the 237 patients, 17 (7.17%) had more than one yeast species, i.e., seven patients were colonized by both *C. albicans* and *C. parapsilosis*; three patients were coinfected with *C. albicans* and *C. glabrata*; two patients had both *C. albicans* and *C. tropicalis*; one patient with *C. albicans* and *C. krusei* (Figure 1); one patient with *C. krusei* and *C. glabrata*; one patient with *C. krusei* and *C. parapsilosis*; and one patient with *C. parapsilosis* and *C. lusitaniae*. One patient had three different *Candida* species, including *C. albicans*, *C. krusei* and *C. glabrata* (Table 1). One isolate of each species recovered from the same patient was analyzed further.

**Table 1:** Distribution of yeast species in VVC patients

Infection status	Number (%)	Yeast species	Number (%)
Single infection	220 (92.83)	*C. albicans*	117 (49.37)
		*C. parapsilosis* complex	57 (24.05)
		*C. glabrata* sensu stricto	24 (10.13)
		*C. tropicalis*	9 (3.80)
		*Pichia kudriavzevii (C. krusei)*	6 (2.53)
		*C. africana*	4 (1.69)
		*Saccharomyces cerevisiae*	2 (0.84)
		*Candida nivariensis*	1 (0.42)
Co-infection	17 (7.17)	*C. albicans* and *C. parapsilosis* complex	7 (2.95)
		*C. albicans* and *C. glabrata*	3 (1.27)
		*C. albicans* and *C. tropicalis*	2 (0.84)
		*C. albicans* and *C. krusei*	1 (0.42)
		*C. krusei* and *C. glabrata*	1 (0.42)
		*C. krusei* and *C. parapsilosis* complex	1 (0.42)
		*C. parapsilosis* complex and *C. lusitaniae*	1 (0.42)
		*C. albicans*, *C. krusei* and *C. glabrata*	1 (0.42)
**Total**	**237 (100)**		**237 (100)**

**Fig. 2:**
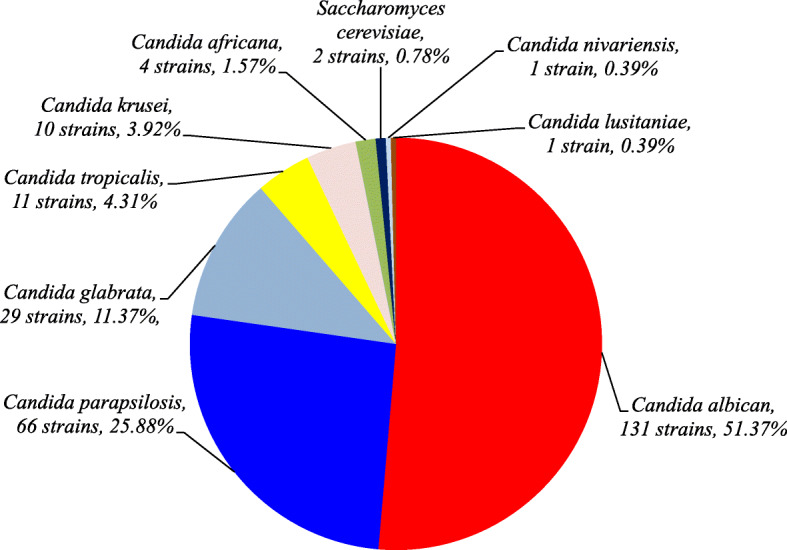
The distribution of the yeast from VVC among non-pregnant reproductive age women

**Fig. 3:**
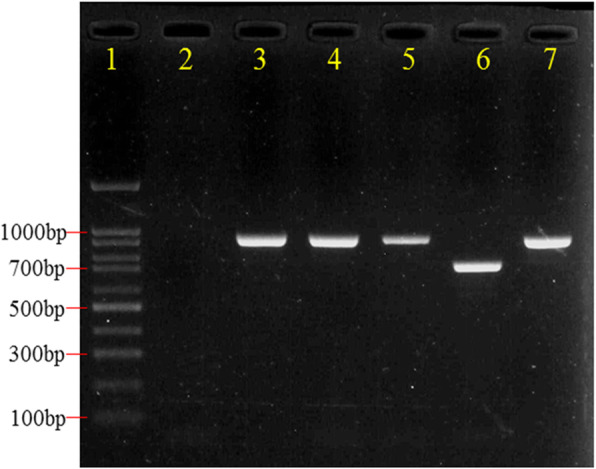
Gel electrophoresis of *C. albicans* complex PCR products targeting the HWP1 gene. Lane 1: molecular size standard (100 bp DNA ladder); lanes 2: negative control; lane 3: positive control (*C. albicans* ATCC 90028); lanes 4, 5 and 7: strains SD106, SD107 and SD158 (*C. albicans,* 940 bp); lane 6: strain SD139 (*C. africana,* 700 bp)

**Fig. 4:**
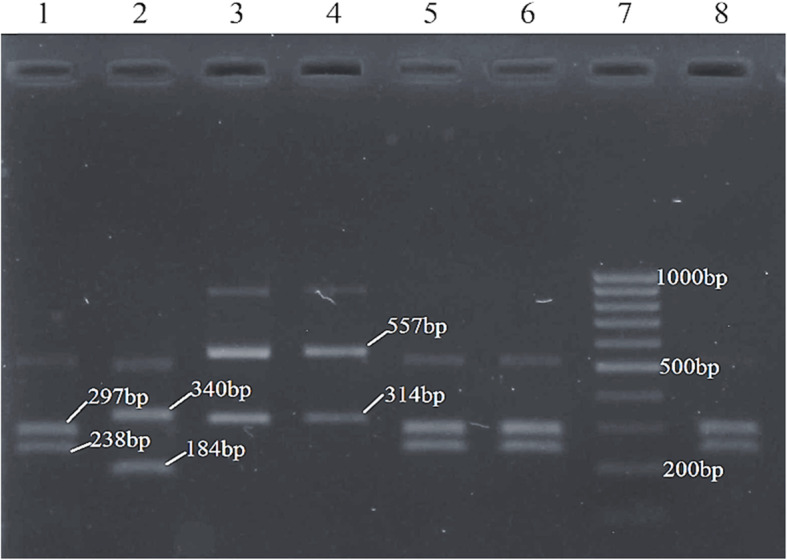
Patterns of PCR products after digestion with the restriction enzyme *Msp*I. Lanes 1, 5, and 6: strains SD1, SD5 and SD6 (*C. albicans*); lane 2: strain SD2 (*C. tropicalis*); lanes 3 and 4: strains SD3 and SD4 (*C. glabrata*); *lane* 7: 100 bp DNA ladder; lane 8: positive control (*C. albicans* ATCC 90028)

A total of 255 yeast isolates were obtained from the 237 vaginal samples. Based on CHROMagar™ Candida medium, PCR, PCR-RFLP, and gene sequencing methods, nine yeast species were identified, the most common yeast was *C. albicans* (131 isolates, 51.37%) followed by *C. parapsilosis* (66 isolates, 25.88%), *C. glabrata* (29 isolates, 11.37%), *C. tropicalis* (11 isolates, 4.31%), *C. krusei* (10 isolates, 3.92%), *C. africana* (4 isolates, 1.57%), *S. cerevisiae* (2 isolates, 0.78%), *C. nivariensis* (1 isolate, 0.39%), and *C. lusitaniae* (1 isolate, 0.39%) (Fig. 2, Fig. 3 and Fig. 4).

The observed susceptibility rates of 46 *C. albicans* isolates to MFG, CAS, AMB, 5-FC, FCZ, ICZ, VCZ and MCZ were 100.0% (46), 95.65% (44), 95.65% (44), 91.30% (42), 91.30% (42), 82.61% (38), 86.95% (40) and 100.0%, respectively. Some drug-resistant isolates were found. This yeast showed 4.35, 6.52 and 4.35% resistance to the FCZ, ICZ, and VCZ, respectively. The resistance rates of *C. albicans* to amphotericine B and 5-Flucytosine were 4.35% (Table 2).

**Table 2:** Ranges of MICs, and percentage resistance in 46 *C. albicans* isolates

Antifungal agents	MIC ranges	MIC_**50**_	MIC_**90**_	MIC_**GM**_	S (%)	I/SDD (%)	R (%)
Micafungin	≤0.015–0.03	≤0.015	0.015	0.015	100.0	0	0
Caspofungin	≤0.03–0.5	0.06	0.25	0.077	95.65	4.35	0
Amphotericine B	0.5–2	1.0	1.0	0.886	95.65	–	4.35
5-Flucytosine	≤0.12–64	0.12	2.0	0.427	91.30	4.35	4.35
Fluconazole	≤0.12–64	0.12	2.0	0.232	91.30	4.35	4.35
Itraconazole	≤0.03–8	0.06	0.25	0.109	82.61	10.87	6.52
Voriconazole	≤0.015–8	0.015	0.25	0.031	86.95	8.70	4.35
Miconazole	≤0.03–8	0.03	0.12	0.043	100.0	0	0

*Note:* MIC_50_: concentration at which 50% of the isolates were inhibited; MIC_90_: concentration at which 90% of the isolates were inhibited. The breakpoints for antifungal agents used for *C. albicans* are: MFG (S ≤ 0.25; I/SDD = 0.5; R ≥ 1); CAS (S ≤ 0.25; I/SDD = 0.5; R ≥ 1); AMB (S ≤ 1; R ≥ 2); 5-FC (S ≤ 4; I/SDD = 8–16; R ≥ 32); FCZ (S ≤ 2; I/SDD = 4; R ≥ 8); VCZ (S ≤ 0.12; I/SDD = 0.25–0.5; R ≥ 1), ICZ (S ≤ 0.12; I/SDD = 0.25–0.5; R ≥ 1) and MCZ (S < 8; R ≥ 8)

*Abbreviation: S* susceptible, *I/SDD* Intermediate/susceptible dose dependent, *R* Resistant

## Discussion

Vulvovaginal candidiasis (VVC) is the second most common vaginal infection in reproductive age women [31]. The results from different studies indicate that the prevalence of vulvovaginal candidiasis varies between countries, depending on the country, region, and population [14, 31–33]. In cases of complicated VVC, vaginal cultures are necessary to confirm clinical diagnosis and identify unusual species, because patients are more likely to have an infection with non-*albicans Candida* strains, which may require different treatment [13, 34] but in Vietnam this issue has received very little attention. Therefore, the current study has been performed to determine the prevalence, species distribution of yeast causing vaginal discharge and antifungal susceptibility pattern of *C. albicans* among symptomatic non-pregnant women of reproductive age in Ha Noi city, Vietnam.

Multiple previous studies showed the prevalence rate of VVC among reproductive age women varies between countries and different regions, ranging from 12 to 72% [32, 35]. In this study, the incidence of VVC in Ha Noi city, Vietnam was found to be 51.30%. Although the prevalence rate of VVC in our study was within the reported range, it was higher than the prevalence rates reported by Anh et al. (1996) [36] that also performed in Ha Noi and Lien et al. (2002) in Hue, central Vietnam [37]. The reasons for such varying prevalence of VVC in Vietnam might be explained the investigation of different geographical locations, profile of the population being studied and period of time in these studies [31]. Therefore, more studies are required to determine the specific rate of VVC in Vietnam.

The availability of epidemiological data from around the world showed that the prevalence rate of VVC among reproductive age women had many differences [5, 32, 35]. The results of our study were similar to that of previous researches in Turkey (49.2%) [38], Egypt (50.4%) [21], Iran (50.5 and 51.6%) [39, 40]. Lower yeast prevalence was reported from Greece (12,1%) [41], UAE (13.88%) [42], India (20.0%) [43], Gabon (28.52%) [44], Ghana (36.5%) [45], Lebanon (39.0 and 44.8%) [1, 33], and Tanzania (45.7%) [46]. Studies from Yemen, Ethiopia reported also a lower prevalence of VVC among non-pregnant reproductive-aged women than the current study [20, 47]. Higher prevalence rates have been reported in Saudi (53.5%) [48], Nigeria (57.3%) [5, 32], and Brazil (72.7%) [35]. Varying prevalence could be due to multiple factors, including socio-demographic characteristics, immune status of patients, treating patients with broad spectrum antibiotics and immune suppressive drugs, and hormonal influences, etc. [20]. VVC affects women globally, and therefore, more studies are required to a better knowledge of the incidence of VVC [5].

Identification of *Candida* species is becoming increasingly important because there has been a notable shift in the etiology of candidiasis with non-*albicans Candida* (NAC) species gaining prominence [9]. In the current investigation, nice yeast species were isolated, including *C. albicans*, *C. parapsilosis*, *C. glabrata*, *C. tropicalis*, *C. krusei*, *C. africana*, *S. cerevisiae*, *C. nivariensis*, and *C. lusitaniae*. Among that, *C. albicans* was the most dominant isolated species (51.37%), whereas the overall prevalence of NAC species was 48.63%. Many previous studies have reported findings that agree with our results, including Guzel et al. (50.4 and 49.6%) [38], Hazirolan et al. (53.9 and 46.1%) [49], and Bitew et al. (58.6 and 41.4%) [20]. The data in different parts of the world have recorded higher rates of *C. albicans* in VVC (75–90%) [7, 39, 42, 44, 50], while lower rates of 25.9, 41.7 and 44.21% were reported from Ghana [51], Pakistan [52] and Iran [17], respectively. According to previous reports, *C. albicans* was responsible for 85–95% of VVC patients; however, most studies, published during the last years, reported incidence of *C. albicans* below 85% and in some regions even below 50% [5]. In fact, the distribution of *Candida* species isolated from women with VVC varies greatly depending on the location as well as the study population [1, 2]. In general, comparing the results of our study with those other studies which demonstrated, our incidence of candidiasis caused by of NAC were quite high. Notably, the most common NAC species in this study are *C. parapsilosis* accounting for 25.88% of all *Candida* species. Our result differs from other studies that implicate *C. grabrata* as the predominant NAC species causing VVC. According to most previous studies, the frequency rates of VVC attributed to *C. glabrata* were around 5–25% of cases [5, 9, 14, 50]. However, other NAC species have been reported as the most prevalent, such as *C. tropicalis* and *C. krusei* in Pakistan [52], *C. krusei* in Ethiopia [20, 53], and *C. famata* in Gabon [44].

Multiple studies have demonstrated the prevalence of *Candida* coinfection among VVC patients varies from 1 to 10% [5]. In the current study, *Candida* coinfection was observed in 17 (7.17%) patients. Lower coninfection prevalence was reported from China (2.2%) [54], Tunisia (3%) [55] and United States (4.8%) [30]. Higher prevalence rates reported in Iran (10.3 and 28%) [17, 56], Australia (13.4%) [57] and Turkey (14.1%) [38]. The prevalence of *Candida* coinfection in Vietnam are not very different from those in other parts of the world. Notably, the majority of the coinfection, 7 out of 17 (41.18%), were *C. albicans* and *C. parapsilosis*. These results of our study were slightly different from reports from around the world. According to previous studies, *C. albicans* and *C. glabrata* co-infection was the most common mixed infection [5, 30, 38]. These differences may be due to the high prevalence of *C. albicans* and *C. parapsilosis.*

The results of this study indicated that *C. albicans* was susceptible to most of the tested antifungals. The resistance rates of *C. albicans* isolates to azoles were lower than 6.25%. Multiple studies around the world also showed good in vitro activity of azoles against *C. albicans* isolates from VVC [20, 22, 58–60]. In contrast, the results previous researches in China [16, 61–63], Ethiopia [53] and Pakistan [52] indicated that susceptibility of *C. albicans* isolates from VVC to azoles were lower than those in other regions. In our study, all the *C. albicans* strains were susceptible to MFG and MCZ. The susceptibility rate of *C. albicans* to CAS was 96.65%. These results have shown that MFG, CAS and MCZ may provide an opportunity for treating azole-resistant VVC. Our results also showed good in vitro activity of AMB against *C. albicans* isolates from VVC. These results were in agreement with previously published reports from China [22, 63], Egypt [21], Lebanon [1] and Iran [39]. According to Philips (2005), amphotericin B vaginal suppositories have been successfully used in cases of azole-resistant *Candida* species [64]. Our findings also showed the prevalence of flucytosine resistance in *C. albicans* isolates were low. However, the speed at which yeast can develop resistance to flucytosine has driven clinicians to use the compound in combination with mainly amphotericin B [65].

## Conclusion

There is a high prevalence of VVC among symptomatic non-pregnant women of reproductive age in Vietnam. Our findings also showed a high incidence of non- *albicans Candida* strains causing vulvovaginitis in the study population, which should be looked at as both novel and alarming. Resistance rates of vaginal *C. albicans* isolates to antifungal agents was low. Extensive surveillance studies of changes in species distribution and antifungal susceptibility should be routinely screened and treated.

## Supplementary Information

**Additional file 1.**

## Data Availability

The datasets used and/or analysed during the current study are available from the corresponding author on reasonable request.

## Abbreviations

AMB: Amphotericin B
ATCC: American Type Culture Collection
CAS: Caspofungin
CLSI: Clinical and Laboratory Standards Institute
FCZ: Fluconazole
GM: Geometric mean
ICZ: Itraconazole
ITS: Internally Transcribed Spacer
MFG: Micafungin
MIC: Minimal Inhibitory Concentration
MCZ: Miconazole
PCR: Polimerase Chain Reaction
RFLP: Restriction Fragment Length Polymorphism
SDA: Sabouraud Dextrose Agar
SDD: Susceptible dose dependent
VVC: Vulvovaginal candidiasis
VCZ: Voriconazole
5-FC: Flucytosine
