# Supplementary material for: Prevalence, species distribution and antifungal susceptibility of Candida albicans causing vaginal discharge among symptomatic non-pregnant women of reproductive age at a tertiary care hospital, Vietnam
Source: BMC Infect Dis. 2021 Jun 3;21:523. doi: 10.1186/s12879-021-06192-7 (PMC8176683; doi:10.1186/s12879-021-06192-7)
Supplement: Supplementary file 1 — Additional file 1. [file 12879_2021_6192_MOESM1_ESM.pdf]

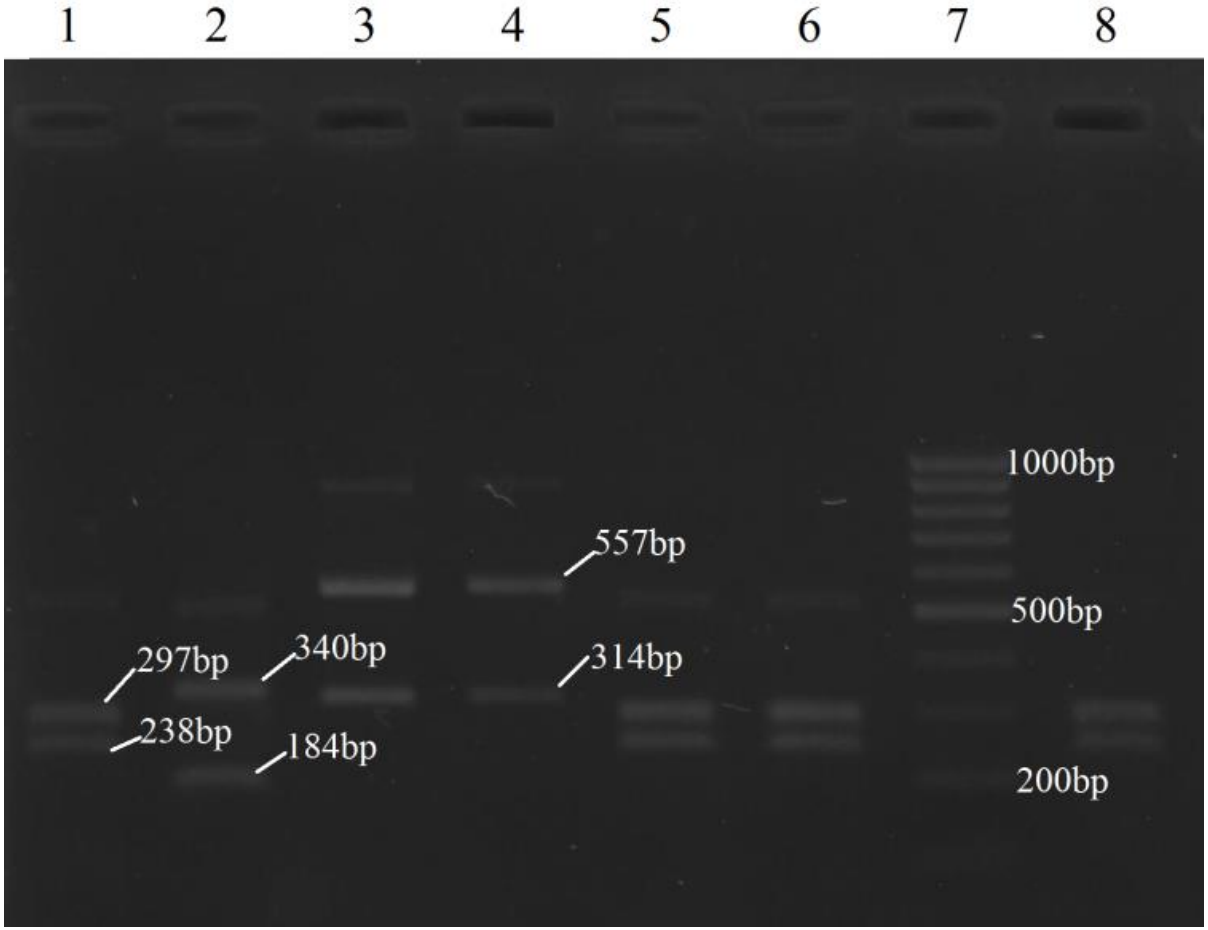

Figure S1. Patterns of PCR products after digestion with the restriction enzyme *Msp*I Lane 1, 5 and 6: *C. albicans* (Strains SD1, SD5, and SD6); lanes 2: *C. tropiclais* (Strains SD2); lanes 3, 4: *C. glabrata* (Strains SD3, SD4); Lane 7: 100 bp DNA ladder; lane 8: Positive control (*C. albicans* ATCC90028)

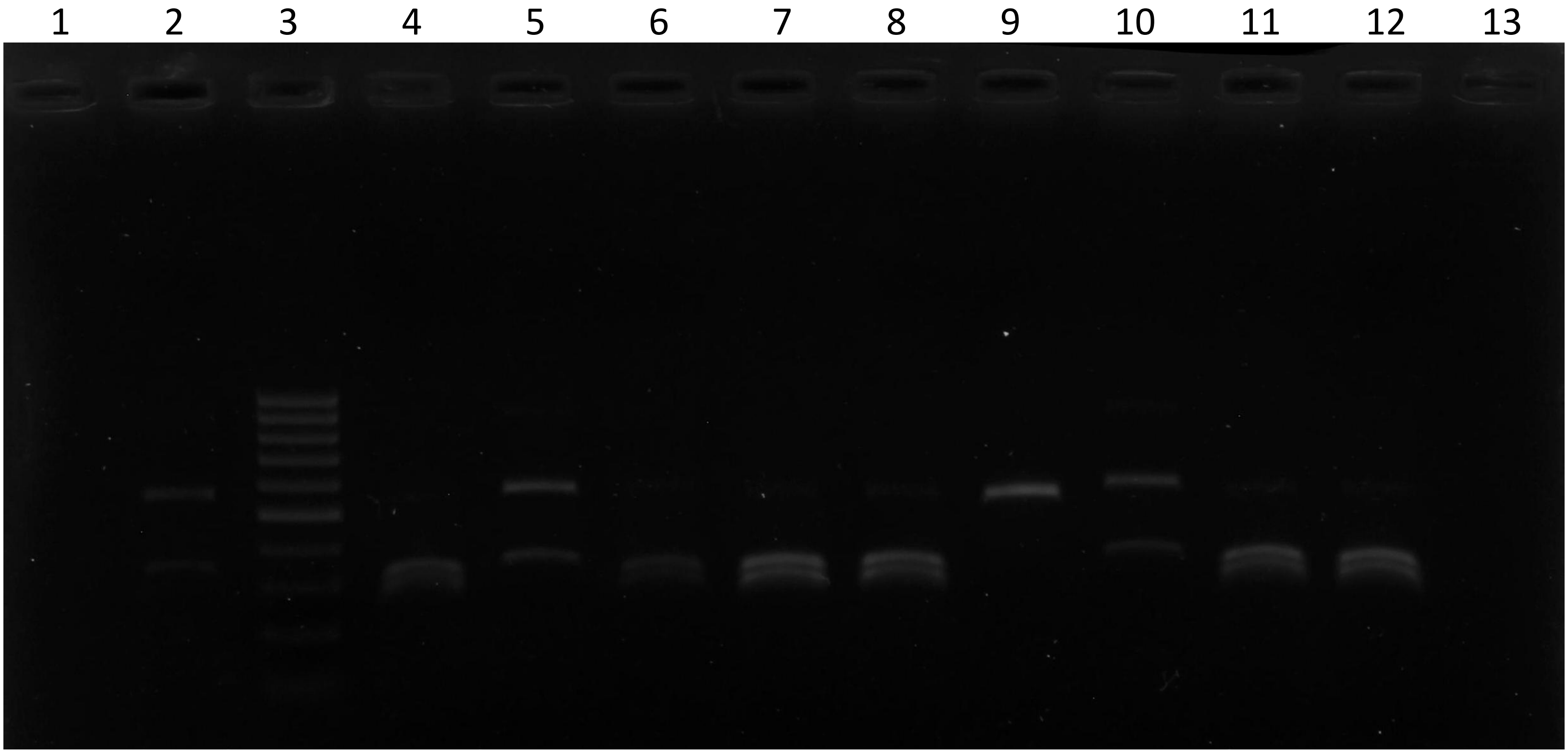

Figure S2. Patterns of PCR products after digestion with the restriction enzyme *Msp*I Lane 1: negative control; lane 2: positive control (*C. glabrata*); lanes 5 and 10: *C. glabrata* (Strains SD4, SD10 and SD10); lanes 4, 6-8 and 11-12: *C. albicans* (Strains SD7, SD11, SD12, SD13, SD17, and SD18); lanes 9: *C. parapsilosis* (Strains SD15); lane 3: 100 bp DNA ladder; lane 13: none

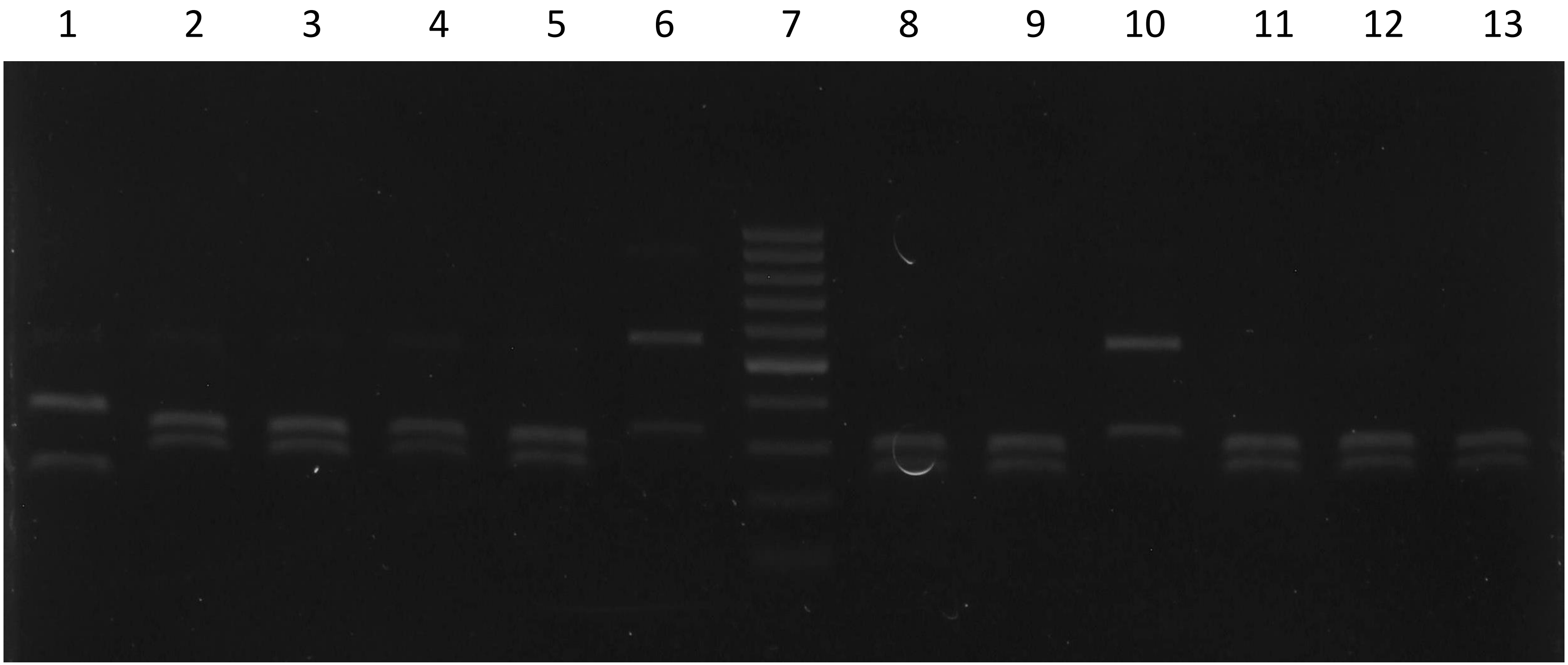

Figure S3. Patterns of PCR products after digestion with the restriction enzyme *Msp*I Lane 1: *C. tropicalis* (Strain SD19); lanes 2-5, 8-9 and 11-13: *C. albicans* (Strains SD20, SD21, SD22, SD23, SD25, SD26, SD28, SD29 and SD30); lanes 6, 10: *C. glabrata* (Strains SD24 and SD27); lane 7: 100 bp DNA ladder

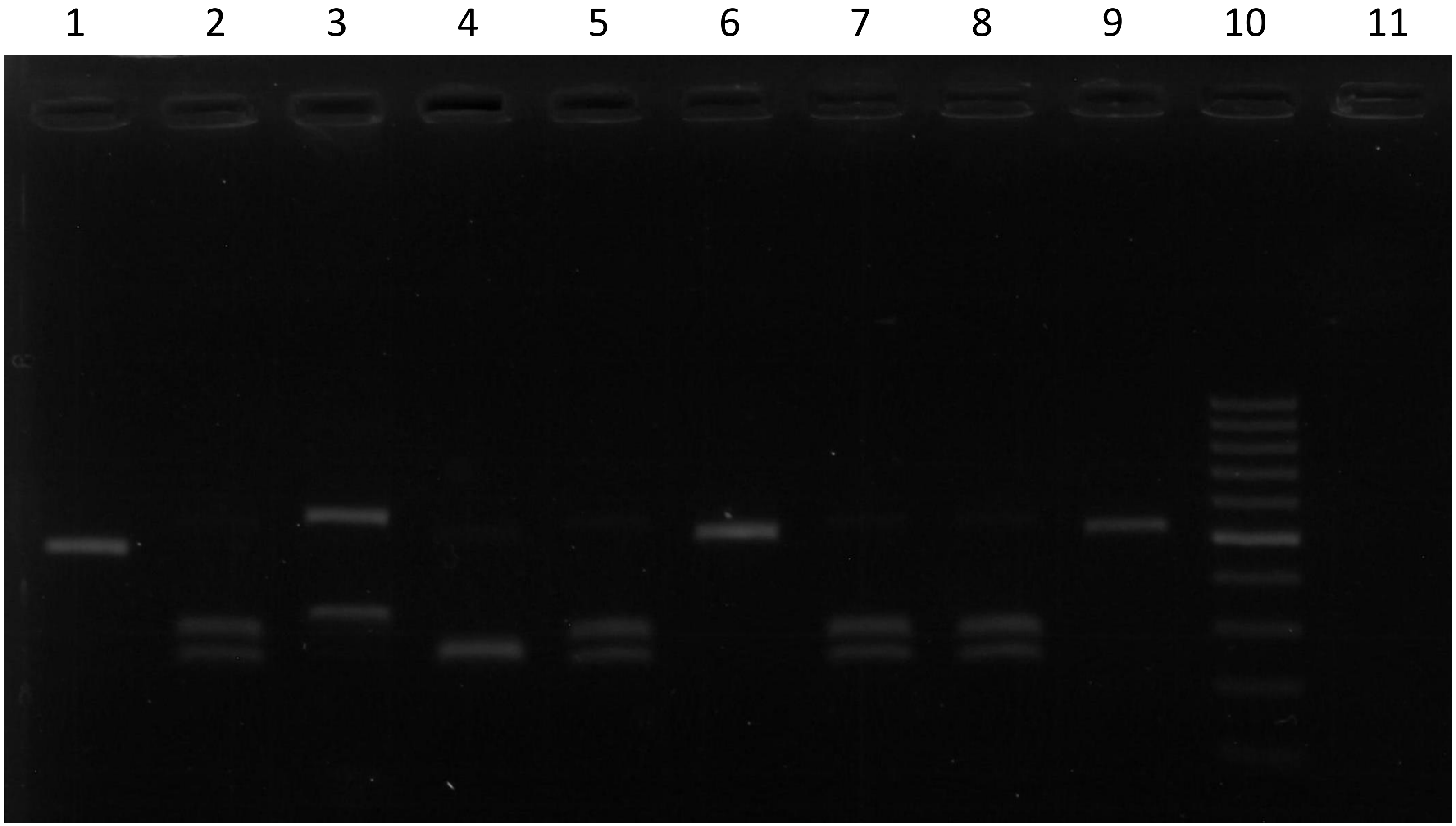

Figure S4. Patterns of PCR products after digestion with the restriction enzyme *Msp*I Lane 1, 6 and 9: *C. parapsilosis* (Strain SD31, SD36 and SD39); lanes 2, 5, 8 and 9: *C. albicans* (Strains SD32, SD35, SD37 and SD38); lane 3: *C. glabrata* (Strains SD33); lane 4: *C. krusei* (strain SD34); lane 10: 100 bp DNA ladder; lane 11: negative control.

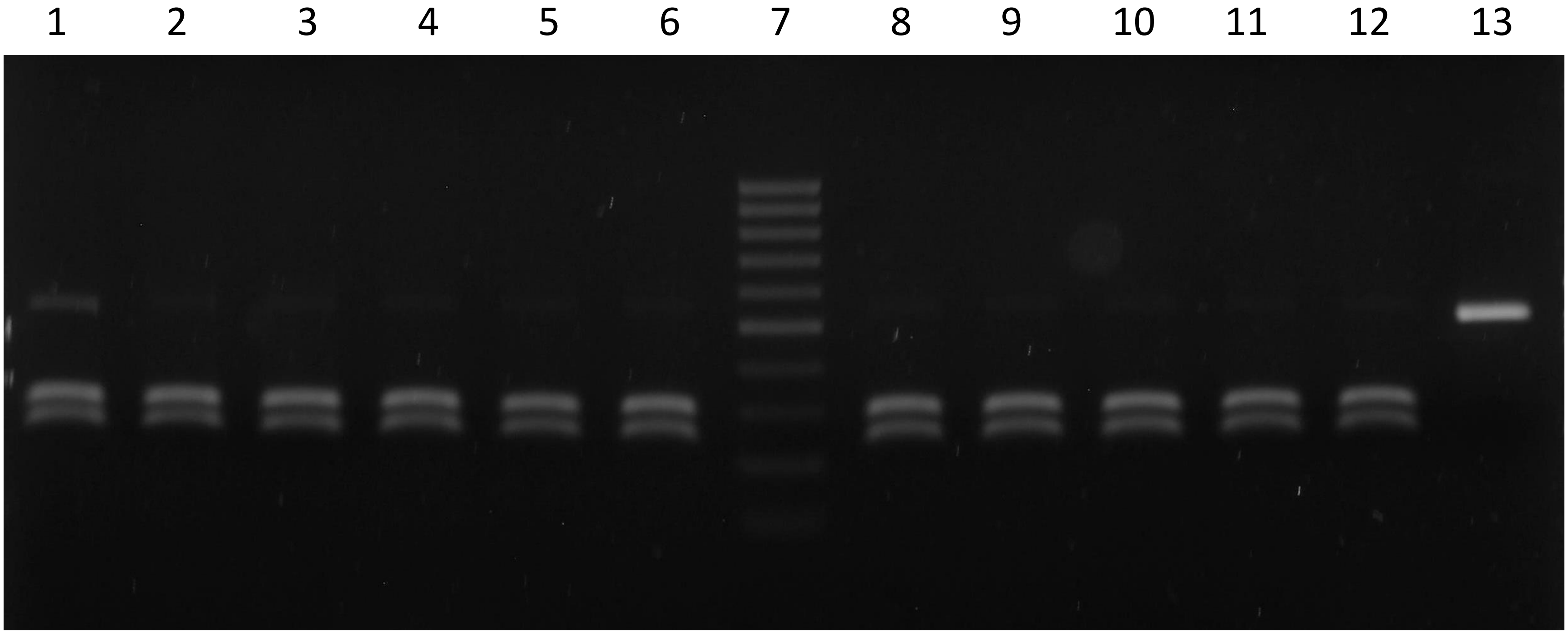

Figure S5. Patterns of PCR products after digestion with the restriction enzyme *Msp*I Lanes 1-6, 8-11: *C. albicans* (Strains SD44, SD62, SD64, SD70, SD74, SD80, SD84, SD85, SD88 and SD97); lane 7: 100 bp DNA ladder; lane 12: Positive control (*C. albicans* ATCC90028), lane 13: Positive control (*C. parapsilosis* ATCC22019),

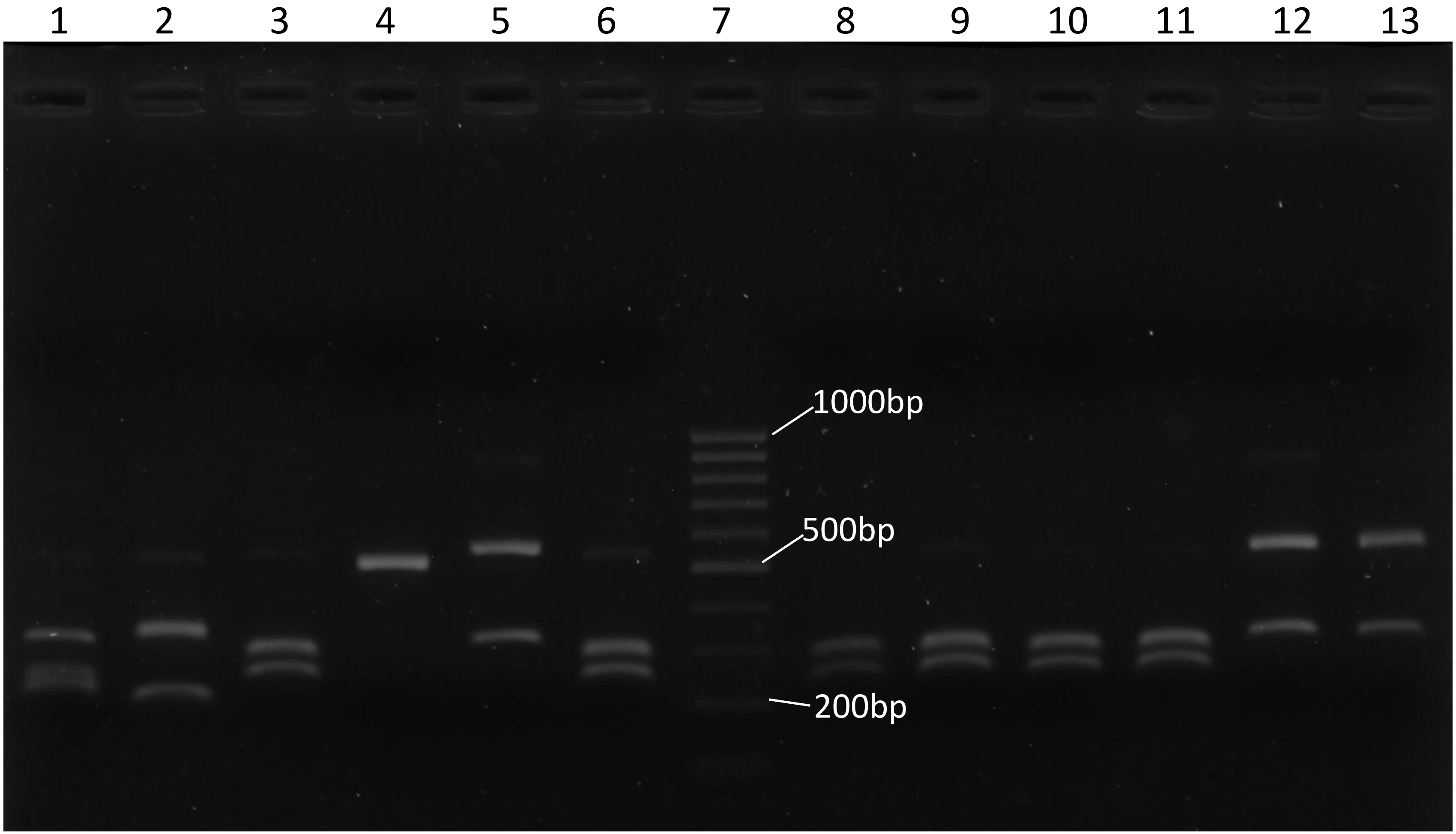

Figure S6. Patterns of PCR products after digestion with the restriction enzyme *Msp*I Lane 1: *not determined* (Strains SD101); lane 2: *C. tropicalis* (Strain SD102); lanes 3, 6, 8-11: *C. albicans* (Strains 103, SD106, SD107-110); lane 4: *C. parapsilosis* (Strain SD104); lanes 5, 12, 13: *C. glabrata* (Strains SD105, SD111 and SD112); lane 7: 100 bp DNA ladder;

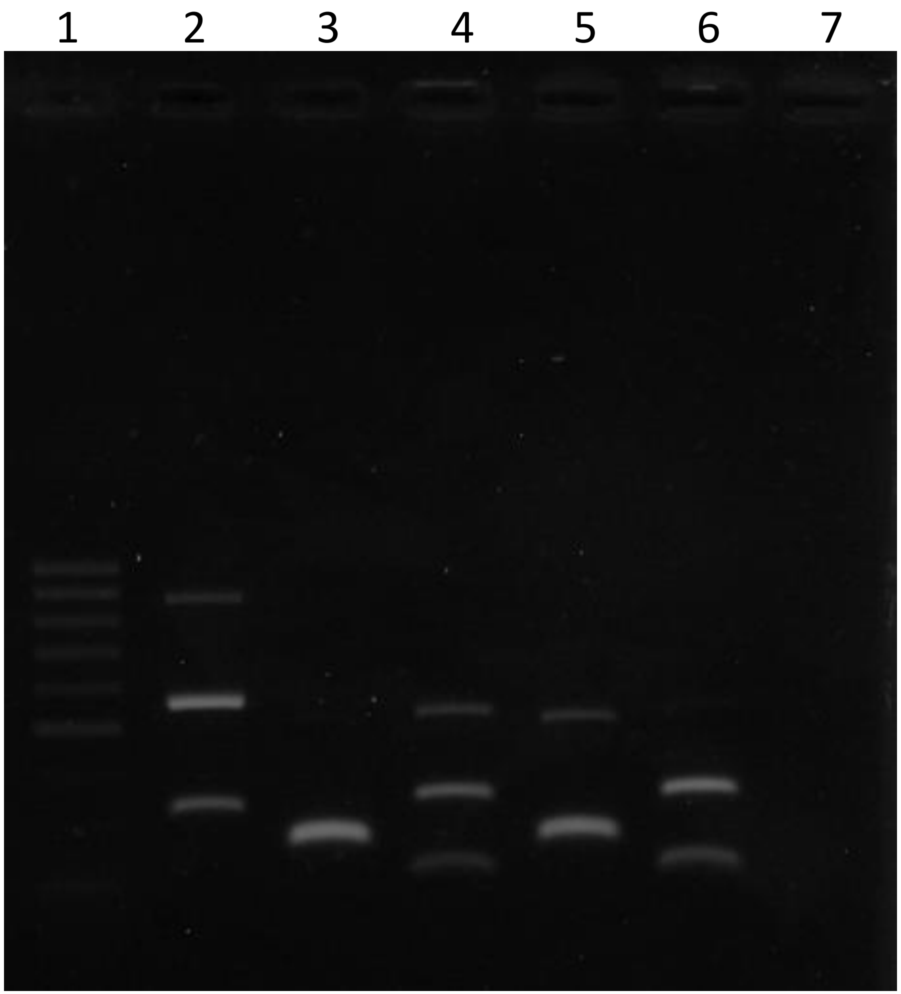

Figure S7. Patterns of PCR products after digestion with the restriction enzyme *Msp*I Lane 1: 100 bp DNA ladder; lane 2: *C. glabrata* (Strain SD173); lanes 3, 5: *C. krusei* (Strains SD178 and SD181); lanes 4, 6: *C. tropicalis* (strains SD180 and SD182); Lane 7: Negative control;

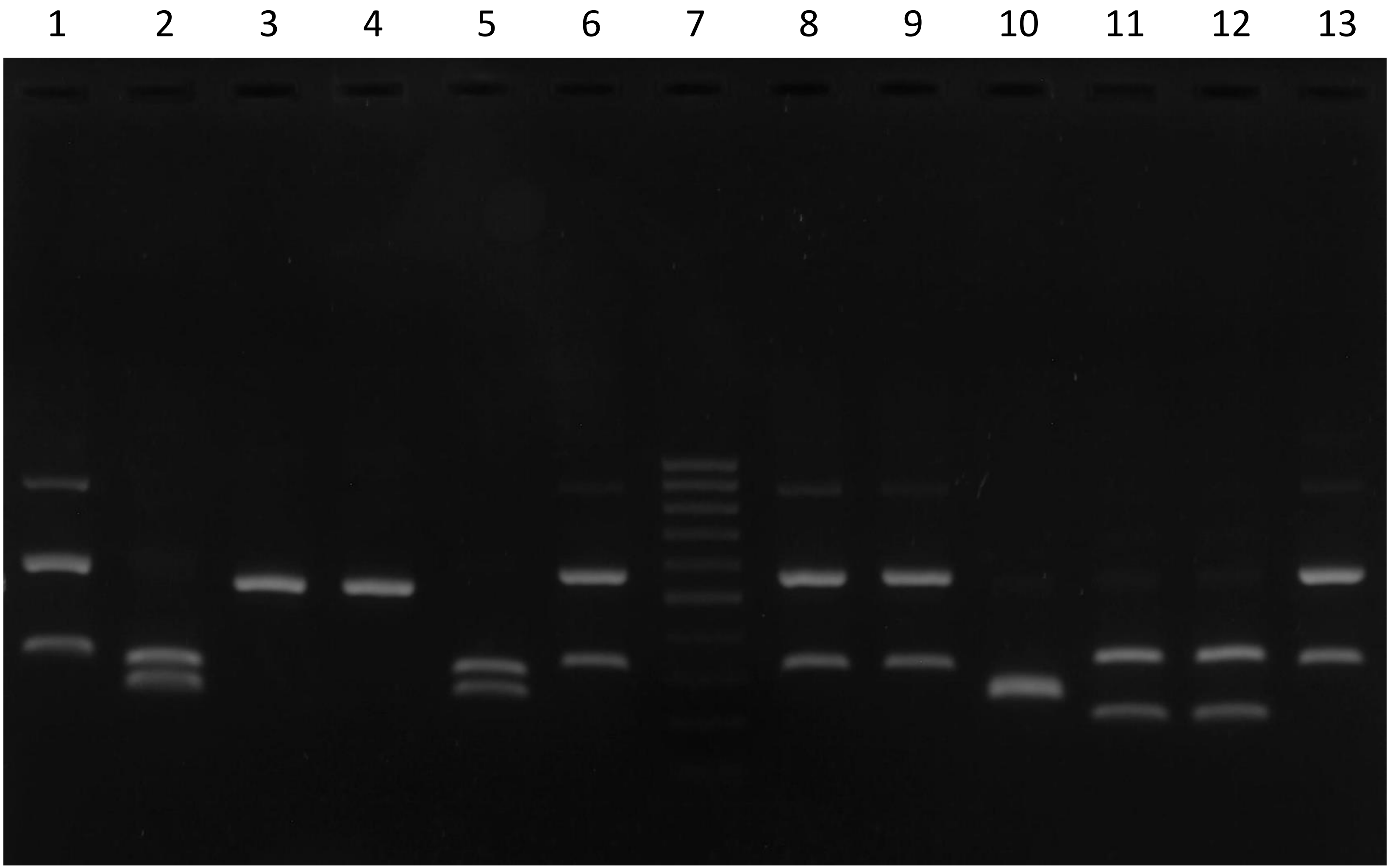

Figure S8. Patterns of PCR products after digestion with the restriction enzyme *Msp*I Lanes 1, 6, 8, 9, 13: *C. glabrata* (Strains SD189, SD197, SD199, SD201, and SD207); lanes 2, 5: *C. albicans* (Strains SD190 and SD196); lanes 3, 4: *C. parapsilosis* (Strains SD194 and SD195); lane 10: *C. krusei* (Strain SD202); lanes 11,12: *C. tropicalis* (Strains SD204 and SD205); Lane 7: 100 bp DNA ladder;

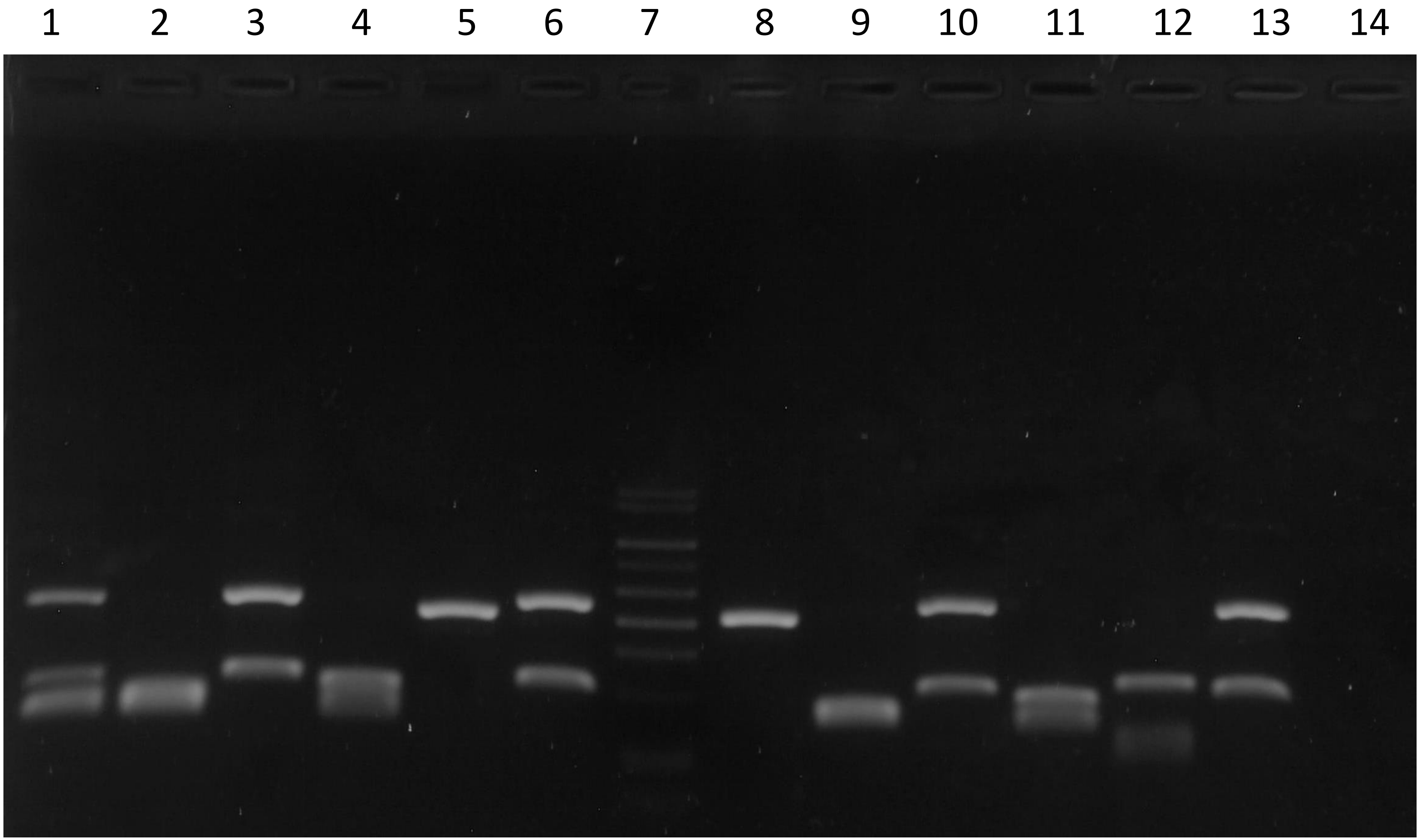

Figure S9. Patterns of PCR products after digestion with the restriction enzyme *Msp*I Lanes 1, 4, 11: *C. albicans* (Strains SD208, SD211 and SD219); lanes 2, 9: *C. krusei* (Strains SD209 and SD216); lanes 3, 6, 10, 13: *C. glabrata* (Strains SD210, SD213, SD217 and SD220); lanes 12: *C. tropicalis* (Strain SD219); Lane 7: 100 bp DNA ladder; lane 14: Negative control

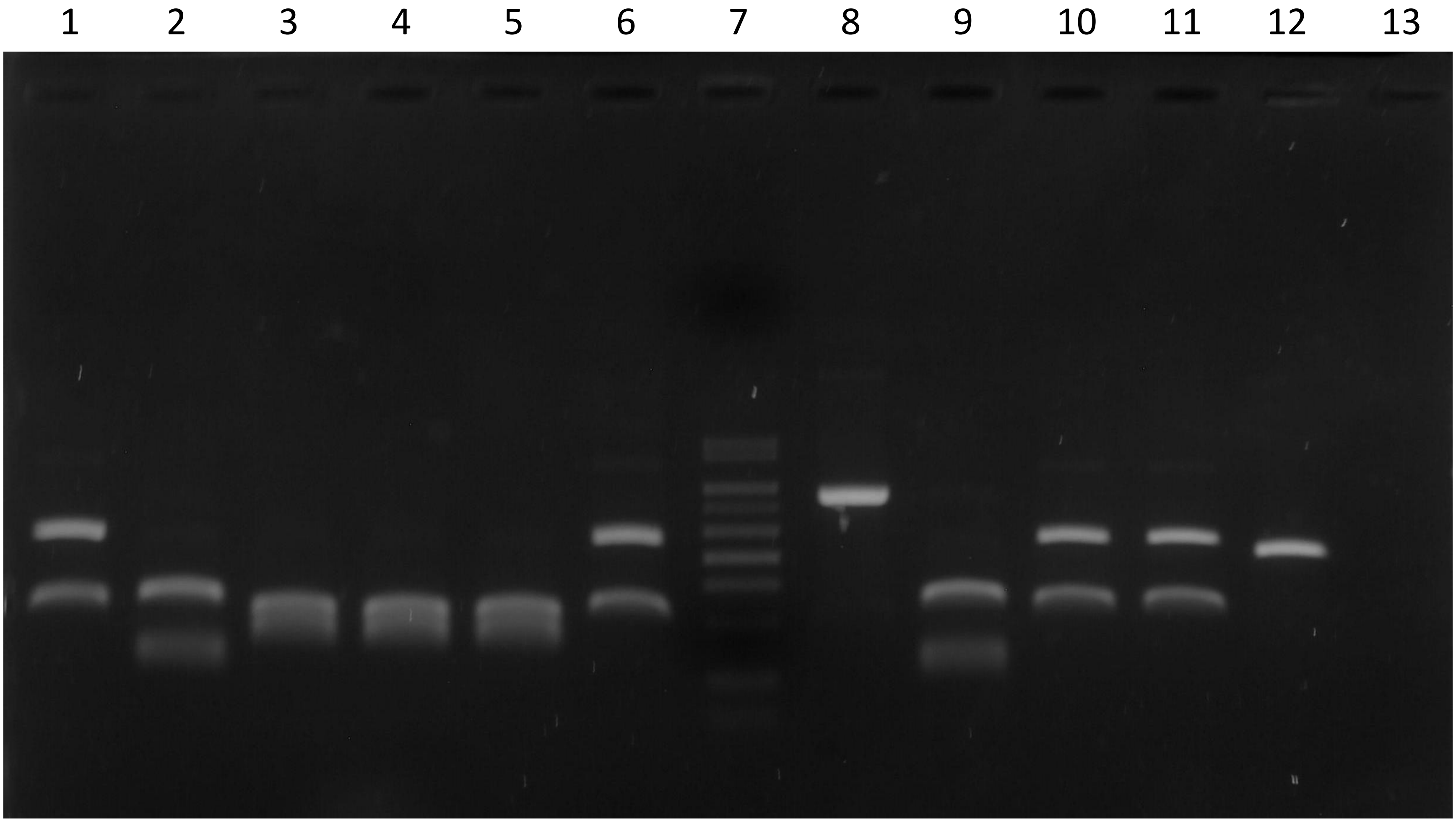

Figure 10. Patterns of PCR products after digestion with the restriction enzyme *Msp*I Lanes 1, 6, 10 and 11: *C. glabrata* (Strains SD221, SD226, SD230 and SD231); lane 2, 9: *C. tropicalis* (Strains SD222 and SD229); lanes 3, 4 and 5: *C. albicans* (Strains SD223, SD224 and SD225); lane 8: not determined; lane 7: 100 bp DNA ladder; lane 12: positive control (*C. parapsilosis* ATCC 22019); lane 13: negative control

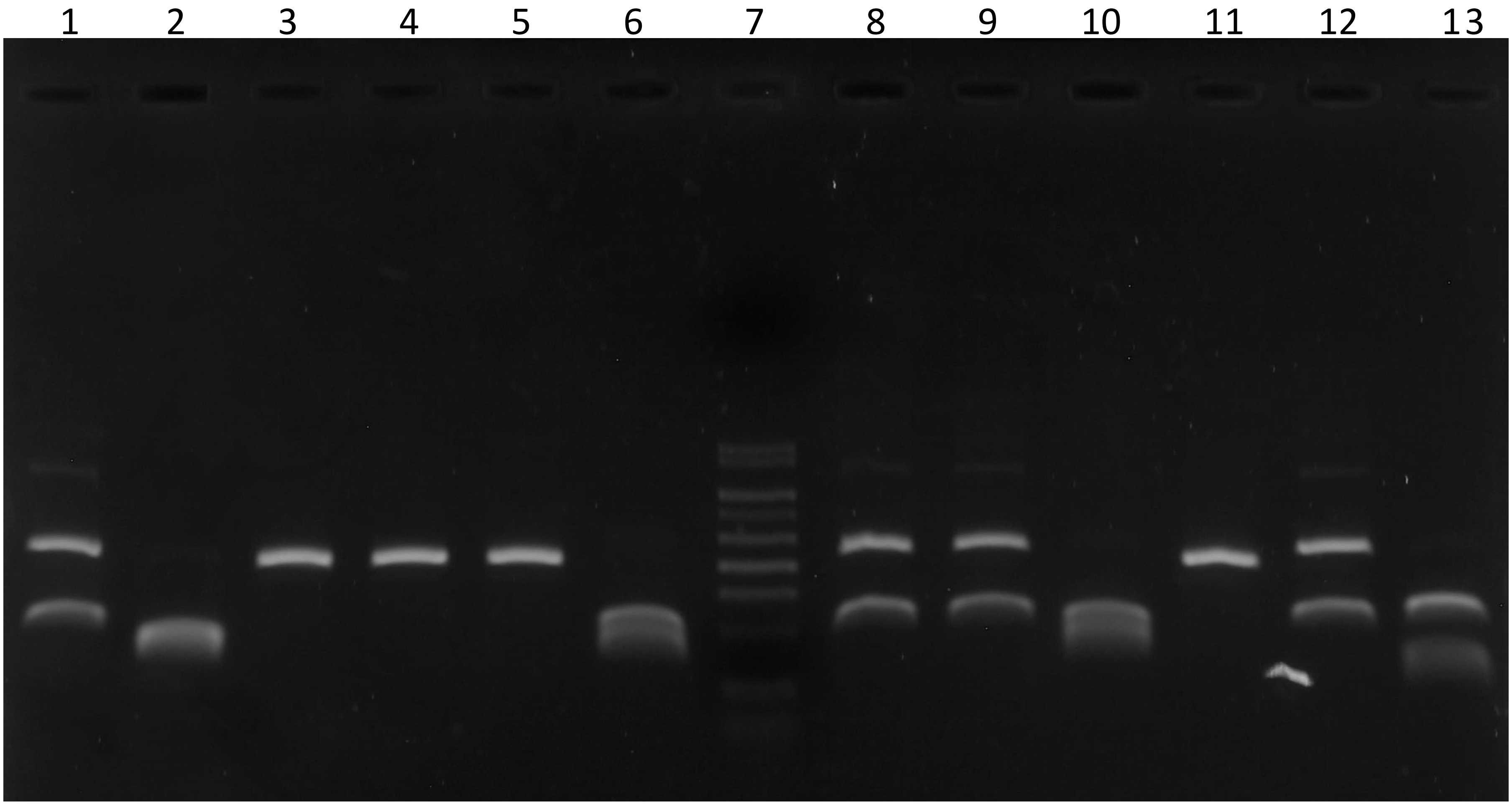

Figure 11. Patterns of PCR products after digestion with the restriction enzyme *Msp*I Lanes 1, 8, 9 and 12: *C. glabrata* (Strains SD233, SD240, SD244 and SD251); lane 2: *C. krusei* (Strains SD234); lanes 3, 4, 5 and 11: *C. parapsilosis* (Strains SD235, SD236, SD238 and SD248); lanes 6, 10: *C. albcians* (Strains SD243 and SD247); lane 7: 100 bp DNA ladder; lane 13: positive control (*C. tropicalis*);
